# Advances in Multi-Omics Research on Biomarkers of Intrahepatic Cholangiocarcinoma

**DOI:** 10.3390/cimb47110905

**Published:** 2025-10-31

**Authors:** Jingxue Yang, Jintong Na, Tieliu Shi, Xiyu Liu

**Affiliations:** 1 State Key Laboratory of Targeting Oncology, Guangxi Medical University, Nanning 530021, Guangxi, China; 202221573@sr.gxmu.edu.cn (J.Y.); najintong@sr.gxmu.edu.cn (J.N.); 2 National Center for International Research of Bio-Targeting Theranostics, Guangxi Medical University, Nanning 530021, Guangxi, China; 3 Guangxi Key Laboratory of Bio-Targeting Theranostics, Guangxi Medical University, Nanning 530021, Guangxi, China; 4 Collaborative Innovation Center for Targeting Tumor Diagnosis and Therapy, Guangxi Medical University, Nanning 530021, Guangxi, China; 5 Guangxi Talent Highland of Major New Drugs Innovation and Development, Guangxi Medical University, Nanning 530021, Guangxi, China

**Keywords:** intrahepatic cholangiocarcinoma, biomarkers, multi-omics analysis, review

## Abstract

Intrahepatic cholangiocarcinoma (iCCA) is a rare yet aggressive malignancy characterized by late-stage diagnosis and poor prognosis. Recent advancements in multi-omics technologies have significantly enhanced the understanding of the molecular features and complex biological mechanisms of iCCA. These approaches have revealed disease molecular heterogeneity and identified biomarkers linked to disease progression, patient prognosis, and therapeutic outcomes. This review synthesizes findings from multi-omics studies, highlighting their clinical implications and advancing the application of precision oncology in iCCA management.

## 1. Introduction

Cholangiocarcinoma (CCA) is a biologically heterogeneous malignancy, anatomically classified into intrahepatic (iCCA), perihilar (pCCA), and distal (dCCA) subtypes [1]. iCCA arises within the intrahepatic bile ducts and may originate from biliary epithelial cells, peribiliary glands, Hering duct progenitor cells, or hepatic stem cells; rare cases involve transdifferentiation from hepatocytes [2]. Clinically, iCCA is staged according to the AJCC TNM system, which considers tumor size, vascular invasion, and metastasis [3]. However, this morphology-based framework fails to reflect the tumor’s molecular complexity, limiting its prognostic and therapeutic utility. In response, recent national and international guidelines have advocated for histological subclassification of iCCA into small-duct and large-duct types [4]. These subtypes differ significantly in cellular origin, molecular alterations, and clinical behavior: small-duct iCCA is frequently associated with IDH1/2 mutations and FGFR2 fusions, whereas large-duct iCCA more commonly harbors KRAS and TP53 mutations [5,6,7]. This histological distinction enhances prognostic stratification and underpins the development of personalized targeted therapies.

The incidence and mortality of iCCA have risen steadily in recent years, positioning it as the second most common primary liver malignancy after hepatocellular carcinoma (HCC), and accounting for approximately 10–20% of all primary liver cancers [8,9]. Its etiology is multifactorial, involving hepatitis B or C, nonbiliary cirrhosis, steatohepatitis, metabolic syndrome, advanced age, bile duct stones, biliary adenomas, Caroli’s disease, choledochal cysts, and parasitic infections [8,10]. Notably, nearly 50% of cases present without identifiable risk factors, and early stages are often asymptomatic, contributing to diagnoses at an advanced stage [3]. Surgical resection remains the only potentially curative treatment option; however, only 20–30% of patients are eligible, and the five-year post-resection survival rate remains low, at 20–35% [3]. These clinical challenges highlight the urgent need for reliable biomarkers to support early detection, prognostic stratification, and therapeutic decision-making.

Biomarkers are defined by the FDA and NIH as indicators that objectively measure and evaluate normal biological processes, pathogenic processes, or responses to therapeutic interventions” [11]. In iCCA, biomarkers help elucidate molecular subtypes, deepen mechanistic understanding, and enable individualized treatment strategies. Advances in molecular biology have accelerated the adoption of multi-omics approaches, including genomics, transcriptomics, proteomics, and metabolomics, that reveal the extensive heterogeneity and molecular complexity of iCCA. These platforms not only facilitate novel biomarker discovery but also form the foundation for refined classification and stratified therapeutic interventions. Nevertheless, many currently proposed biomarkers remain limited by inadequate sensitivity, specificity, and clinical applicability, largely due to pronounced tumor heterogeneity, insufficient external validation, and a lack of standardized detection methodologies [12,13].

This review presents a comprehensive overview of iCCA biomarkers identified through multi-omics strategies, emphasizing their current roles and limitations in early detection, molecular classification, prognostic assessment, and individualized therapy. Drawing on recent integrative analyses, we highlight biomarker combinations with translational promise and outline future directions to advance precision medicine in iCCA.

## 2. Molecular Biomarkers at Different Omics Levels

**Figure 1:**
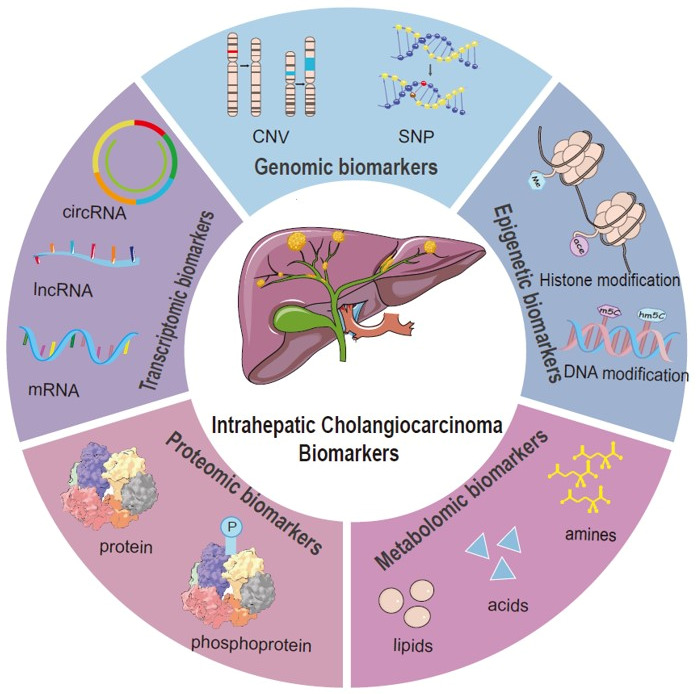
Overview of research directions for intrahepatic cholangiocarcinoma biomarkers across multi-omics levels.

Recent advances in multi-omics technologies have enabled comprehensive analyses of the molecular characteristics of iCCA through genomics, transcriptomics, proteomics, metabolomics, and epigenomics, leading to the identification of biomarkers linked to the disease onset, progression, and prognosis (Figure 1).

### 2.1. Genomic Biomarkers

Genomics aims to elucidate the role of genetic alterations—including mutations, deletions, fusions, and amplifications—in tumor initiation and progression by investigating genome composition, structure, function, and gene–environment interactions. The advent of next-generation sequencing technologies, such as whole-genome sequencing (WGS) and whole-exome sequencing (WES), has enabled comprehensive profiling of the iCCA genome, significantly advancing insights into its molecular landscape [13].

Commonly identified mutations in iCCA include inactivating alterations in IDH1/2, ARID1A, TP53, BAP1, PBRM1, and ATM, as well as activating mutations in KRAS, BRAF, NRAS, RASA1, TERT, and NF1 [14,15]. These somatic mutations are frequently regarded as oncogenic drivers central to tumorigenesis and disease progression. Structural variations such as FGFR2 and ROS1 fusions, CDKN2A/B deletions, and amplifications in MET, ERBB2, EGFR, PBX1, c-MET, FGF19, CDK6, and CCND1 have also emerged as key genomic events in iCCA [15,16,17]. These alterations are often accompanied by chromosomal copy number variations (CNVs), including gains in 1q, 6q, 7p, 11q, and 12q, and losses in 3p, 6q, 8p, 9p, 10q, 12p, 12q, 13q, 14q, 16q, 19p, and 21q [18,19]. Collectively, these findings highlight the extensive genomic heterogeneity of iCCA and underscore the relevance of genetic alterations in influencing diagnosis, therapeutic response, and clinical outcome, while identifying candidate targets for precision oncology.

Notably, patterns of mutual exclusivity have been observed among key mutations—for instance, between TP53 and IDH1, IDH1 and KRAS, TP53 and BAP1, and IDH1 and FGFR2 [17]. These mutually exclusive relationships suggest that distinct mutations may drive divergent oncogenic pathways, resulting in unique molecular phenotypes and differential therapeutic susceptibilities [20]. Moreover, mutation prevalence appears to vary by race and sex, with FGFR2 mutations being more common in African American patients, and IDH1, FGFR2, and BAP1 mutations more frequently observed in females [21,22]. These population-specific patterns further support the development of personalized treatment strategies.

iCCA can be histologically classified into small-duct and large-duct subtypes, which have been shown by genomic studies to exhibit distinct mutational landscapes [5,7]. The small-duct subtype is typically characterized by IDH1/2 mutations and FGFR2 fusions, along with widespread alterations in metabolism-related genes, observed in approximately 10–30% of cases, and is generally associated with more favorable clinical outcomes [7]. In contrast, large-duct iCCA more frequently harbors KRAS and TP53 mutations—found in 15–30% of patients—and is linked to increased tumor aggressiveness and poorer prognosis [6]. Identification of these molecular alterations facilitates not only subtype stratification but also informs therapeutic decision-making (Table 1). Targeted therapies have shown promise in select molecular contexts. Tumors harboring IDH1/2 mutations have shown responsiveness to ivosidenib (AG-120) [23], while FGFR2 fusions are associated with an objective response rate of 35.5% to pemigatinib [24,25]. By contrast, KRAS-targeted therapies, such as sotorasib and adagrasib, have demonstrated efficacy in lung and colorectal cancers but show limited benefit in iCCA [26,27]. Similarly, vemurafenib yields a modest response rate of 12.5% in BRAF-mutated iCCA, underscoring the lack of effective treatment strategies for non-IDH/FGFR-driven subtypes [28].

Genomic approaches have demonstrated clear advantages in identifying genetic mutations and susceptibility loci in iCCA [26]. However, their inherently static nature limits the capacity to capture gene expression dynamics under varying physiological and pathological conditions. Compared with transcriptomic, proteomic, and metabolomic analyses, genomics alone offers limited insight into tumor microenvironment interactions or individualized therapeutic responses [29]. To address these limitations, future research should focus on integrating genomic data with other omics layers to achieve a more comprehensive understanding of the functional consequences of genetic alterations in iCCA.

**Table 1:** Key Genomic Biomarkers with Therapeutic Potential and Corresponding Targeted Agents in iCCA.

Acronym (Full Name)	Frequency [14,15]	Oncogenic Alterations	Biologic Function	Targeted Therapy Drugs [30,31]
IDH1/2 (isocitrate dehydrogenase)	14–36%	mutation	encoding enzymes involved in the citric acid cycle	ivosidenib/AG-120, AB-218 capsule (NCT 5814536-Ⅰ), dasatinib (NCT 2428855-Ⅱ), IDH305 (NCT 2977689-Ⅰ)
FGFR2 (Fibroblast growth factor receptor 2)	10–15%	fusion	encoding a receptor tyrosine kinase involved in cell growth and angiogenesis	Pemigatinib/INC054828, Infigratinib/BGJ398 (NCT 3773302-Ⅲ), Derazantinib/ARQ087 (NCT 3230318-Ⅱ), TT-00420 (NCT 4919642-Ⅱ), HMPL453 (NCT 4353375-Ⅱ), HH185/D185 (NCT 5039892-Ⅱ), Lenvatinib/E7080 (NCT 02579616/04211168-Ⅱ), Gunagratinib (NCT 3758664-Ⅰ/Ⅱ), E7090 (NCT 4238715-Ⅰ), RLY-4008 (NCT 4526106-Ⅰ/Ⅱ)
EGFR (epidermal growth factor receptor)	25%	amplification	encoding a receptor tyrosine kinase that regulates cell proliferation and survival	erlotinib, geitinib, cetuximab, panitumumab
BRAF (B-Raf proto-oncogene, serine/threonine kinase)	5–7%	mutation	encoding a kinase involved in the RAS/MAPK pathway	Vemurafenib, dabrafenib
KRAS (Kirsten rat sarcoma viral oncogene homolog)	7–22%	mutation	encoding a GTPase, playing a key role in the RAS/MAPK signaling pathway	Trametinib, Selumetinib
BAP1 (breast cancer 1-associated protein 1)	10–15%	mutation	encoding a deubiquitinating enzyme that regulates chromatin and DNA repair	romidepsin, vorinostat, valproic acid
PBX1 (Pre-B-cell leukemia homeobox transcription factor 1)	29.20%	amplification	a transcription factor that regulates numerous embryonic processes including hematopoiesis	Nonclinical drugs
ERBB3 (Erb-B2 Receptor Tyrosine Kinase 3)	7%	amplification	encoding a member of the EGFR family, involved in cellular growth and differentiation	Nonclinical drugs

### 2.2. Transcriptomic Biomarkers

Transcriptomics offers a powerful framework for comprehensively characterizing gene transcription and regulatory mechanisms at the cellular level. Widely employed techniques include bulk RNA sequencing (bulk RNA-seq) and single-cell RNA sequencing (scRNA-seq). Bulk RNA-seq enables population-level analysis of gene expression patterns, whereas scRNA-seq provides high-resolution insights into intratumoral heterogeneity and discrete cellular subpopulations [32,33]. Spatial transcriptomics (ST), though still in its infancy within the context of iCCA, represents a promising tool for mapping gene expression in situ [34]. Comparative transcriptomic analyses of tumor, peritumoral, and adjacent normal tissues in iCCA have revealed numerous subtype-specific transcriptional biomarkers. These markers hold substantial promise for enhancing diagnostic precision, refining prognostic stratification, and informing the development of targeted therapeutic strategies (Table 2).

#### 2.2.1. mRNA Biomarkers

A recent scRNA-seq study identified MAL2 as highly enriched in malignant cholangiocytes in iCCA, implicating it as a key driver of tumorigenesis [35]. Elevated MAL2 expression is associated with increased proliferation and poor prognosis, likely mediated by activation of the EGFR/SREBP-1 axis, which reprograms lipid metabolism and promotes cell migration and invasion [35]. The small-molecule inhibitor sarizotan significantly suppresses tumor growth and enhances cisplatin sensitivity in vitro and in vivo [35]. Initially developed for non-oncologic indications and currently in clinical trials (e.g., NCT02790034), sarizotan offers repurposing potential in oncology due to its established pharmacokinetic and safety profile [49]. However, its efficacy and specificity in iCCA require further preclinical and clinical validation. Given its role in chemoresistance and the availability of a candidate inhibitor, MAL2 represents a promising therapeutic target in drug-resistant iCCA. In a separate scRNA-seq study, two histological subtypes of iCCA were identified, with S100P and SPP1 serving as representative markers of tumor cells derived from large and small bile ducts, respectively [50]. These expression patterns were consistently observed across patients, supporting their utility in molecular classification and personalized management.

MFAP5 has also emerged as a promising mRNA biomarker with both diagnostic and prognostic relevance. By modulating the Notch pathway, it promotes tumor cell invasiveness, and elevated expression is significantly associated with poor survival [36]. Importantly, serum MFAP5 levels can distinguish iCCA patients from healthy controls, highlighting its potential as a non-invasive screening biomarker. It also shows higher specificity and sensitivity than conventional markers like CEA and CA19-9 and declines significantly post-surgery, suggesting a role in monitoring treatment response and disease progression [36]. Nevertheless, clinical translation of MFAP5 as a liquid biopsy marker requires further validation to ensure feasibility, reproducibility, and stability in real-world settings.

Overexpression of HES1 has been shown to induce the formation of proliferating ductal cells (PDCs), contributing to iCCA pathogenesis. PDCs are regarded as “opportunistic” hepatic progenitor-like cells with stem-like features that may serve as a cellular origin of iCCA under specific pathological conditions [51]. Accordingly, HES1 has been proposed as a therapeutic target in PDC-derived iCCA. However, the cellular origin of iCCA is highly heterogeneous, and the precise contribution of PDCs remains unclear, rendering the therapeutic value of targeting HES1 uncertain. Furthermore, no HES1 inhibitors have yet reached clinical development, limiting its translational feasibility. Thus, despite its mechanistic relevance, HES1 currently has limited practical utility as a therapeutic target.

#### 2.2.2. LncRNA Biomarkers

Long non-coding RNAs (lncRNAs) contribute to iCCA pathogenesis through diverse mechanisms, including interactions with functional proteins, regulation of competing endogenous RNA (ceRNA) networks, activation of signaling pathways, and epigenetic modulation of gene expression. Several lncRNAs, such as MNX1-AS1, ZEB1-AS1, SPRY4-IT1, and CASC15, are overexpressed in iCCA tissues and significantly associated with poor overall survival (OS) and progression-free survival (PFS) [38,39,52,53]. These transcripts, typically identified in tumor specimens, partially reflect tumor aggressiveness and may serve as prognostic biomarkers. However, current evidence largely derives from invasive, tissue-based analyses. Non-invasive lncRNA biomarkers detectable in accessible body fluids, such as blood, remain underexplored, limiting their integration into routine clinical practice and broader translational use.

Beyond broadly prognostic lncRNAs, certain transcripts may enable more nuanced risk stratification. lnc-CDK9-1 and HOXC13-AS are significantly upregulated in patients with poor prognosis, whereas lnc-CBLB-5 and COL18A1-AS2 are enriched in those with favorable outcomes [54]. These lncRNAs may serve as supplementary markers for individualized risk assessment, though their incremental predictive value over existing models requires validation in prospective, multicenter studies.

In addition to their prognostic relevance, several lncRNAs have shown therapeutic potential. Preclinical studies have provided initial evidence of functional roles for specific lncRNAs in iCCA. For example, CASC15 knockdown inhibits proliferation and migration while inducing apoptosis [39]; PAICC silencing markedly reduces xenograft tumor growth [55]; and FAM66C promotes glycolysis and invasiveness via the miR-23b-3p/KCND2 axis [37]. These findings highlight select lncRNAs as candidate therapeutic targets in iCCA. However, further functional validation and delivery strategies are required for clinical translation.

#### 2.2.3. CircRNA Biomarkers

Circular RNAs (circRNAs) are covalently closed, single-stranded RNAs generated via back-splicing of precursor mRNAs. Their high stability, evolutionary conservation, and tissue- or stage-specific expression have attracted growing interest in cancer research. CircRNAs have demonstrated broad potential in diagnosis, prognostic assessment, and therapeutic intervention.

In iCCA, multiple circRNAs exhibit dysregulated expression and hold diagnostic and prognostic value due to their tissue specificity and subtype stratification capabilities. For example, circACTN4 overexpression correlates with shorter three-year overall survival and higher recurrence risk, while circHMGCS1-016 independently predicts recurrence following curative resection [44,45]. circCCAC1, upregulated in CCA tissues and detectable in bile-derived exosomes, outperforms conventional serum markers diagnostically and is linked to poor pathological features across all CCA subtypes, serving as an independent predictor of prognosis and recurrence in iCCA [46]. circLTBP2 is highly expressed in iCCA but minimally in HCC, underscoring strong tissue specificity. Its serum detectability highlights potential utility as a non-invasive biomarker [43].

CircRNAs also exhibit functional potential in targeted therapy by modulating drug response pathways and non-coding RNA networks. For example, silencing circZNF215 enhances the efficacy of the AKT inhibitor ipatasertib, whereas circPCNXL2 overexpression diminishes the antitumor effect of the MEK inhibitor trametinib [40,42]. In contrast, circNFIB overexpression may delay trametinib resistance, suggesting a role in combination therapy [56]. circPKM promotes paclitaxel resistance by sponging miR-199a-5p and upregulating STMN1 [57]. To counteract this, a dual-delivery nanoplatform combining si-circPKM with paclitaxel significantly improves treatment efficacy [57]. Another circRNA, circUGP2, is downregulated in iCCA and inversely correlated with advanced TNM stage. Lipid nanoparticle-mediated delivery of circUGP2 has demonstrated clear preclinical antitumor activity in vivo [47]. While these findings support the therapeutic promise of circRNAs, most evidence remains confined to cell lines and animal models, with limited validation using clinical specimens. Additionally, key challenges—including delivery biosafety, tissue specificity, and pharmacodynamic persistence—must be addressed before clinical translation can advance.

Beyond drug response, circRNAs also regulate the tumor microenvironment and immune processes. circSLCO1B3 promotes tumor growth and metastasis in iCCA by enhancing immune evasion [41]. circGGNBP2 drives malignant remodeling of the immune microenvironment through sustained IL-6/STAT3 signaling and independently predicts poor prognosis [58]. circRAPGEF5 is involved in SUMOylation-associated tumor regulation, while circFOXP1 and circMBOAT2 influence iCCA progression by modulating metabolic reprogramming and lipid metabolism, respectively [59,60,61]. Although these findings highlight the potential of circRNAs as immunotherapeutic or metabolic targets, most mechanistic insights lack validation in clinical patient cohorts. The functional roles and translational relevance of circRNAs remain incompletely defined, underscoring the need for further mechanistic investigation and clinical correlation.

Collectively, circRNAs serve multifaceted roles in iCCA, functioning as diagnostic, prognostic, therapeutic, and immunometabolic biomarkers. However, current research remains largely limited to preclinical models and early-stage mechanistic studies. Advancing clinical translation will require the establishment of standardized detection platforms, validation in patient cohorts, and the development of safe and efficient delivery systems.

#### 2.2.4. Small RNA Biomarkers

Advancements in high-throughput transcriptomic technologies have intensified interest in the functional and clinical significance of small RNAs, including microRNAs (miRNAs), PIWI-interacting RNAs (piRNAs), tRNA-derived small RNAs (tsRNAs), and small nucleolar RNAs (snoRNAs). These molecules regulate cancer initiation, progression, and treatment response through mechanisms such as signaling modulation, epigenetic regulation, and mRNA degradation [62]. Among them, miRNAs are the most extensively studied and remain the primary focus of small RNA research in iCCA.

In iCCA, miRNAs exhibit notable diagnostic and prognostic potential. For instance, loss of miR-10 upregulates EZH2 expression and enhances tumor invasiveness, suggesting utility in early detection [53]. miR-141-3p and miR-27a-3p promote migration and invasion via Hippo pathway regulation and have been explored as biomarkers for early screening [55]. miR-502-5p, detectable in liquid biopsy samples, is implicated in immune evasion and shows promise as a non-invasive biomarker [41]. From a prognostic perspective, miR-338-3p downregulation correlates with shorter OS and RFS [48], while miR-3191-5p suppresses tumor progression via the ADGRB1/p53 axis and is strongly linked to patient survival [47]. Reduced levels of miR-1236-3p are associated with an unfavorable immune microenvironment and poor prognosis, and miR-199a-5p downregulation correlates with paclitaxel resistance and increased tumor aggressiveness [44,57]. Although several miRNAs are detectable in body fluids such as blood, key technical barriers remain before clinical application. These include insufficient validation of their dynamic stability, specificity, and detection thresholds in clinical settings.

miRNAs also hold therapeutic potential. For example, restoring miR-766-3p expression suppresses tumor growth by modulating the ERK signaling pathway, and reintroduction of miR-3191-5p significantly inhibits tumor dissemination [42,47]. However, the extensive target gene networks and complex regulatory architecture of miRNAs contribute to bidirectional effects, complicating their integration into standardized therapeutic frameworks. Moreover, their variable expression across tissues and microenvironments poses challenges to therapeutic consistency and safety.

Beyond miRNAs, other small RNA species—including piRNAs, tsRNAs, and snoRNAs—remain largely unexplored in iCCA. Although systematic mechanistic studies and clinical data are lacking, early evidence from other cancers suggests translational promise. For instance, snoRNA-associated drug response prediction is supported by the GPSno database, and both piRNAs and tsRNAs have demonstrated diagnostic and therapeutic relevance in colorectal and hepatocellular carcinomas, respectively [63,64,65]. These findings imply that continued advances in functional and spatial transcriptomics may uncover additional small RNA candidates for iCCA biomarker development. Nonetheless, major barriers—including limited tissue specificity, poor molecular stability, and unresolved mechanisms—must be addressed through foundational and translational research.

Overall, transcriptomics has significantly advanced our understanding of iCCA pathogenesis by revealing dynamic gene expression patterns and regulatory networks, which cannot be fully captured by genomics or proteomics alone. This omics layer offers unique advantages in dissecting tumor heterogeneity and defining molecular subtypes, providing a robust foundation for biomarker discovery and therapeutic research. However, most transcriptomic biomarkers remain at the preclinical or mechanistic validation stage, and none have yet been applied clinically as single or panel biomarkers for diagnosis or prognosis. Clinical translation faces significant challenges. Notably, non-invasive RNA biomarkers such as circRNAs and miRNAs encounter technical and pre-analytical limitations, including variability in RNA extraction efficiency, inherent molecular instability, inconsistent normalization methods, and batch effects affecting reproducibility across studies. Future research should focus on standardized sample processing, enhanced RNA stability, and rigorous normalization strategies to facilitate reliable clinical application of transcriptomic biomarkers.

### 2.3. Proteomics Biomarkers

Proteomics has provided critical insights into the molecular mechanisms of iCCA by profiling protein expression and post-translational modifications. Recent advances in high-throughput mass spectrometry have expanded its applications in identifying key protein biomarkers, characterizing subtype-specific differences, and exploring therapeutic targets. A prominent subfield, phosphoproteomics, captures phosphorylation events as dynamic indicators of signaling activity [66]. This approach is particularly useful for evaluating kinase activation, predicting treatment response, and elucidating drug resistance mechanisms.

Using quantitative proteomics, Guo et al. identified distinct expression patterns of glycolysis- and gluconeogenesis-related enzymes across iCCA subtypes. Glycolytic enzymes such as TPI1, GAPDH, and PGK1 were significantly downregulated in large-duct compared to small-duct iCCA, suggesting that metabolic reprogramming contributes to subtype differentiation [67]. These differentially expressed proteins not only aid in molecular classification but also provide a rationale for metabolism-targeted therapies.

In a phosphoproteomic analysis of 52 iCCA samples, four phospho-subtypes (PHOS1-PHOS4) were delineated based on kinase activation profiles. The PHOS2 subtype was enriched for metabolism-associated kinases such as PDK1 and hypoxia-responsive phosphoproteins, and correlated with poor clinical outcomes. In PHOS3, increased phosphorylation of PLK1 was observed—a kinase shared with HCC that promotes tumor progression via metabolic and cell cycle–related pathways. The phosphorylation states of PDK1 and PLK1 are closely linked to metabolic reprogramming, suggesting that dynamic kinase modifications may underlie tumor heterogeneity in iCCA [68]. However, most findings to date are derived from mass spectrometry–based screening with limited functional validation, and the clinical stability and feasibility of these phospho-biomarkers remain to be fully assessed. The study further proposed that enriched phospho-domains may represent convergence hubs of multiple oncogenic signaling pathways, offering novel avenues for subtype classification and targeted intervention. Future therapeutic strategies may benefit from incorporating PLK1 inhibitors (e.g., volasertib) or PI3K/PDK1-targeted approaches in selected subtypes [68].

Beyond phosphorylation, other post-translational modifications, such as lactylation, have gained increasing attention. Using LC-MS/MS, Yang et al. identified nucleolin (NCL) as a key lactylation substrate. Lactylation of NCL upregulated MADD (mitogen-activated protein kinase–activating death domain protein) expression and promoted malignant phenotypes in iCCA cells [69]. These findings indicate that diverse modification mechanisms collectively drive tumor progression and may yield novel biomarkers and therapeutic targets.

Proteomics has also been extensively applied to characterize protein alterations in the iCCA tumor microenvironment, particularly in bile and serum. Aberrant expression of clusterin (CLU) and ferritin in bile has been closely linked to biliary tract malignancies and shows promising diagnostic value [70]. While conventional serum markers such as CEA and CA19-9 are widely used, their specificity remains limited. Combining bile- and serum-derived protein markers may improve diagnostic accuracy [71]. In the context of recurrence, proteomic analyses have identified several prognostic indicators. For example, elevated TPI1 expression is significantly associated with higher recurrence risk, and ST3GAL1, a glycosylation-modifying enzyme, influences metastatic potential—both serving as potential tools for risk stratification [72,73]. Remodeling of extracellular matrix (ECM) proteins plays a critical role in iCCA invasion and metastasis. Upregulation of thrombospondin 1 (THBS1), THBS2, and pigment epithelium–derived factor (PEDF) in tumor tissues creates a soluble anti-angiogenic environment that suppresses blood vessel formation while promoting lymphangiogenesis [74]. The expression profiles of these proteins not only elucidate iCCA invasion mechanisms but also represent candidate targets for microenvironment-focused therapeutic strategies.

The clinical utility of proteomics is further underscored in distinguishing iCCA from HCC. Studies have revealed significant differences between the two malignancies in lipid metabolism and extracellular matrix–related pathways. A biomarker panel comprising apolipoprotein E (APOE), pyruvate kinase L/R (PKLR), and galactokinase 1 (GALK1) was developed to differentiate iCCA from HCC [75]. Validated in an independent cohort, this model demonstrated high diagnostic accuracy and offers a theoretical basis for precise clinical discrimination.

Compared with other omics-derived biomarkers, proteomic markers offer a more direct readout of cellular functional states and metabolic activity. In particular, the dynamic expression and post-translational modification of key enzymes provide insights into the regulation of complex signaling pathways [76,77]. The advent of spatial proteomics further enables simultaneous assessment of protein localization and abundance, thereby enhancing drug target identification and improving the precision of molecular diagnostics and targeted therapy.

Despite these advantages, the clinical translation of protein biomarkers faces several challenges, including inter-patient variability, lack of technical standardization, and limited reproducibility across laboratories. Most current studies are primarily limited to preclinical settings, with a scarcity of large-scale prospective data and limited integration with underlying biological mechanisms [78,79]. To overcome these barriers, future research should prioritize integrative analyses combining proteomic, genomic, and transcriptomic data; promote the development of innovative technologies; and strengthen multicenter collaborations. These efforts are essential to advancing the clinical application of proteomic biomarkers in the diagnosis, classification, and personalized treatment of iCCA.

### 2.4. Metabolomic Biomarkers

Metabolomics, following genomics and proteomics, focuses on detecting small-molecule metabolites in body fluids or tissues to explore their relationships with physiological and pathological changes [80]. In iCCA research, metabolomics offers distinct advantages. Structural lesions in the liver and bile regions directly affect the production and metabolic pathways of metabolites, which are often reflected in the metabolic profiles of liver and bile fluids, such as blood and bile.

For example, Tan et al. conducted a targeted metabolomics analysis of plasma metabolites in iCCA patients and identified 10 metabolites significantly associated with survival, including Valyl-Lysine, quercetin, 12-gingerol, 3-methyl-2-oxovaleric acid; several lysophospholipids (e.g., LysoPC(18:1(9Z)), LysoPC(26:0)); and phosphatidylcholine (PC) [81]. Using these metabolites, a LASSO-Cox predictive model was constructed, which demonstrated high accuracy (AUC > 0.85) in assessing one-year OS following curative resection, suggesting its potential clinical value in iCCA prognosis [81]. Zhang et al. reported elevated glutamine levels in the plasma of iCCA patients compared with healthy controls, supporting its potential as a diagnostic biomarker [82]. A study analyzing serum metabolites in iCCA patients via ultraperformance liquid chromatography–tandem mass spectrometry (UPLC-MS/MS) identified six circulating amino acids—L-asparagine, L-methionine, L-phenylalanine, L-tyrosine, kynurenine, and L-cystine—as potential diagnostic biomarkers [83], leading to the development of a highly sensitive and accurate diagnostic model for iCCA.

Bile, as a liver- and bile-specific fluid, is closely associated with the tumor site and serves as a crucial carrier of metabolites, providing detailed insights into the tumor microenvironment. Studies have shown that iCCA patients have significantly lower total bile acid concentrations and deoxycholic acid ratios in bile than those with benign biliary diseases. Additionally, Shi et al. reported that elevated histamine (HA) levels in the bile of CCA patients are linked to poor prognosis and induce distinct oncogenic effects depending on HA receptor expression profiles [84].

Recent advances in tumor metabolomics have identified R-2-hydroxyglutarate (R-2-HG) as a significantly elevated metabolite in IDH-mutant iCCA. R-2-HG plays a mechanistic role in tumorigenesis and has emerged as a promising therapeutic target. A novel mIDH1 inhibitor, HH2301, designed to suppress R-2-HG activity, has demonstrated superior efficacy in IDH-mutant iCCA patients compared to AG-120—an earlier-generation inhibitor—showed only modest benefit in iCCA clinical trials [85]. These findings underscore the therapeutic potential of R-2-HG as a metabolic target in iCCA.

Metabolomics shows high diagnostic efficacy in distinguishing iCCA from other biliary diseases via combinatory models of metabolites, including carbohydrates, amino acids, and lipids. Unlike other omics approaches, metabolomics emphasizes functional aspects, directly reflecting dynamic biochemical processes. This helps reveal tumor-specific metabolic characteristics and their impact on the surrounding environment [86]. As a result, metabolomics offers greater sensitivity and specificity in detecting early biochemical changes, making it particularly suitable for personalized diagnostics and precision medicine.

### 2.5. Epigenetic Biomarkers

Epigenomics provides new insights into the molecular characteristics of iCCA through the identification of epigenetic changes, including DNA methylation, histone modification, and non-coding RNA regulation. Disrupted DNA methylation patterns are prominent in iCCA. Using whole-genome bisulfite sequencing (WGBS), researchers identified four distinct methylation subtypes (S1–S4), each associated with distinct gene mutation profiles and postoperative prognosis [87]. Studies using droplet digital PCR (ddPCR) to detect DNA methylation markers in bile, such as CDO1, CNRIP1, SEPT9, and VIM, have shown the ability to identify early-stage iCCA up to 12 months before conventional imaging diagnosis [88]. This approach has notable predictive value, particularly in high-risk populations such as those with primary biliary cirrhosis.

The role of N6-methyladenosine (m^6^A) methylation and associated proteins, such as IGF2BP1 and YTHDF2, in RNA regulation has gained increasing attention in iCCA research. Studies have shown that IGF2BP1 stabilizes the expression of multiple oncogenes through m6A-dependent modifications, promoting tumor progression. Additionally, m6A-mediated upregulation of the HLF gene enhances the malignant phenotype of iCCA by activating the FZD4/β-catenin signaling pathway, suggesting the therapeutic potential of IGF2BP1 and the m6A modification pathway [89].

Histone methylation, including H3K9 demethylation and H3K4 methylation, plays a key role in the epigenetic regulation of iCCA. Wang et al. identified significant regulatory relationships between the Kdm6b, Jmjd1C, and Ash1l genes and AP1 target genes (Jun, Fos, Junb) via weighted gene co-expression network analysis (WGCNA) and protein–protein interaction (PPI) networks [90]. These findings suggest that increased H3K9 demethylation and H3K4 methylation may drive iCCA cells into a stress-response subtype. Similarly, interactions between H3K9 trimethylation (H3K9me3), histone deacetylase 1 (HDAC1), and HP1α regulate the STAT1 signaling pathway, influencing iCCA invasion and metastasis [91]. Additionally, METTL3-mediated H3K4 trimethylation promotes iCCA proliferation and metastasis by regulating IFIT2 mRNA degradation in a YTHDF2-dependent manner [92]. METTL5-mediated 18S rRNA m6A modification controls iCCA cell growth and migration [93]. These studies provide valuable insights into tumor progression and resistance mechanisms in iCCA, offering new directions for targeted therapies.

Although significant progress has been made in the epigenetic study of intrahepatic cholangiocarcinoma, the dynamic and reversible nature of epigenetic modifications, along with their sensitivity to environmental factors and the tumor microenvironment, may lead to variability in detection results, posing challenges to their use as reliable biomarkers. Nevertheless, epigenomics offers new insights into the mechanisms of tumorigenesis, progression, migration, and invasion in iCCA. When integrated with other omics approaches, this approach provides critical theoretical support for a comprehensive understanding of iCCA, identifying molecular subtypes, and advancing precision therapies.

## 3. Application of Biomarkers

**Figure 2:**
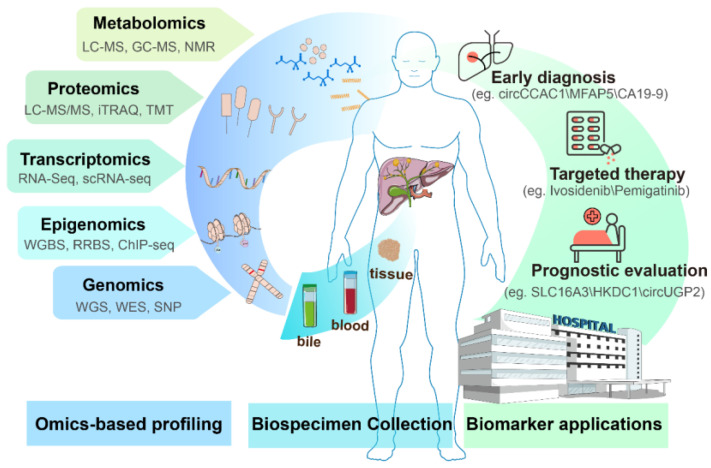
A multi-omics-based framework for biomarker application in iCCA.

A wide spectrum of omics-derived biomarkers has been explored across multiple biological layers to support various clinical decision-making needs in iCCA. As shown in Figure 2, the biomarker development pipeline spans from omics-based discovery to biospecimen collection and clinical translation, with practical applications in diagnosis, therapy selection, and outcome prediction.

### 3.1. Diagnostic Biomarkers

Diagnostic biomarkers are defined as biological indicators used to detect or confirm disease presence or to distinguish between disease subtypes [94]. The diagnosis of iCCA requires a comprehensive assessment of clinical presentation, imaging, laboratory findings, and pathological evaluation. However, conventional serum markers such as CA19-9 and CEA demonstrate limited sensitivity and specificity, complicating the differentiation of iCCA from other hepatobiliary disorders [71]. To enhance diagnostic accuracy, recent studies have shifted toward integrating multi-omics biomarkers into multidimensional composite models, thereby addressing the limitations of single-marker approaches.

An ideal diagnostic biomarker for iCCA should meet the following criteria: (1) enable non-invasive detection via liquid biopsy (e.g., blood); (2) offer sufficient sensitivity for early-stage diagnosis; (3) demonstrate high specificity through tissue- or cell-type–restricted expression; and (4) be cost-effective and suitable for broad clinical implementation [95,96]. Based on current evidence, this review highlights two composite biomarker panels with high translational relevance (Table 3). The first panel integrates glutamine and MFAP5 with conventional protein markers CA19-9 and CEA, spanning metabolic, transcriptomic, and proteomic layers—all detectable in serum [36,71,82]. This combination is well-suited for early screening and longitudinal monitoring. The second panel comprises bile-derived circ-CCAC1 and DNA methylation markers CDO1 and SEPT9, which have shown high specificity and sensitivity in high-risk populations (e.g., patients with primary sclerosing cholangitis) and may serve as auxiliary tools in cases with inconclusive imaging [46,88]. These two panels are complementary in terms of sample source, mechanistic breadth, and clinical utility, providing a feasible foundation for developing non-invasive or minimally invasive precision diagnostic systems for iCCA.

### 3.2. Development of Therapeutic Targets

The role of biomarkers in guiding therapeutic strategies for iCCA has garnered growing recognition, evolving from mutation-based target identification to encompass treatment sensitivity prediction and combinatorial therapy design. Although gemcitabine plus cisplatin remains the standard first-line treatment for advanced iCCA, its clinical benefit is modest, with a median overall survival of less than one year [97]. Targeted therapies, such as FGFR and IDH inhibitors, have shown promise; however, acquired resistance represents a major clinical hurdle, limiting long-term efficacy. Mechanisms of resistance include secondary mutations, activation of bypass signaling pathways, and compensatory feedback loops. Addressing these challenges requires integrative strategies that combine multi-omics profiling with longitudinal patient monitoring, enabling early detection of resistance mechanisms and informing the design of more effective combinatorial treatments.

To improve therapeutic precision, research has shifted from single-target approaches to multidimensional combination strategies. Several biomarker panels have demonstrated strong translational potential at the preclinical stage (Table 4). For example, a metabolic biomarker combination involving IDH1/2 mutations, R-2-HG accumulation, and elevated ALDH expression may identify patients eligible for treatment with mIDH1 inhibitors and ALDH inhibitors such as NCT-501 [23,68,85]. Another promising approach targets the immunosuppressive subtype, defined by KRAS mutations, Treg infiltration, and high PD-1/PD-L1 expression, which may benefit from a combination of KRAS inhibitors (e.g., sotorasib) and immune checkpoint inhibitors [26,27,68].

In addition, early-stage studies have identified several non-coding RNAs that modulate treatment sensitivity in iCCA. For instance, MAL2, circLTBP2, and circHMGCS1-016 have shown potential as therapeutic targets in response to cisplatin, gemcitabine, and PD-1 blockade, respectively [35,43,44]. These biomarkers may serve as adjunctive biomarkers in future combinatorial treatment regimens, supporting the stratification of drug-resistant subgroups and refinement of therapeutic pathways.

### 3.3. Prognostic Biomarkers

Prognostic biomarkers are essential for evaluating patient survival risk, recurrence probability, and postoperative treatment response, and thus are critical to postoperative management and personalized therapeutic decision-making in iCCA. Several individual molecular biomarkers—such as circUGP2, lncRNA CASC15, and miR-338-3p—have demonstrated preliminary utility in reflecting tumor biology and predicting patient outcomes [39,47,48]. However, due to the high molecular heterogeneity of iCCA, single biomarkers are often insufficient to fully capture the disease’s clinical progression.

A more promising approach involves integrating multi-omics data and applying systems biology methods, such as unsupervised clustering, to stratify patients and identify clinically relevant molecular subtypes. Dong et al. established four distinct iCCA subtypes by integrating proteomic, phosphoproteomic, transcriptomic, and genomic profiles (Table 5). These subtypes demonstrated not only multilayered molecular differences but also significant variation in OS [98]. Based on this classification, the study further identified several stable and representative prognostic biomarker panels, providing a robust foundation for postoperative risk assessment and precision surveillance strategies.

## 4. Limitations

Despite significant advancements in biomarker research for iCCA, several limitations should be acknowledged. First, most of the biomarkers discussed in this review originate from exploratory studies with small sample sizes and single-center designs, lacking systematic validation across multicenter cohorts and ethnically diverse populations. This limitation hampers the assessment of their stability and generalizability in real-world clinical contexts [35,42]. Second, although several studies have applied machine learning or deep learning to develop diagnostic, prognostic, or recurrence prediction models, the absence of standardized performance metrics, validation workflows, and implementation frameworks restricts their clinical utility and reproducibility [81,83,99]. Furthermore, the integration of multi-omics data continues to face substantial technical and methodological challenges, including sample heterogeneity, platform inconsistencies, and insufficient batch effect correction—all of which undermine result reproducibility and cross-study comparability. Finally, as a literature-based review, this study did not perform original systematic analyses such as biomarker interaction networks, co-expression modules, or multi-omics clustering, which constitutes a key limitation of the current study. Future research should prioritize the use of systems-level bioinformatics approaches—including network modeling, multimodal clustering, and machine learning—to explore and validate the mechanistic interactions among biomarkers across different molecular layers.

## 5. Discussion and Future Prospects

This review provides a systematic summary of recent progress in biomarker research for iCCA across five key biological dimensions: genetic mutations, transcriptional expression, protein functional states, metabolic characteristics, and epigenetic regulation. These findings, spanning multiple molecular levels, have significantly expanded our understanding of iCCA mechanisms and laid a foundation for biomarker-driven precision medicine.

Building on the existing literature, we propose a novel logic-integrated strategy for constructing biomarker panels. This strategy incorporates upstream genetic alterations, midstream transcriptional regulation, and downstream protein or metabolic pathways into a unified framework. It emphasizes functional complementarity and consistency of sample sources among selected biomarkers. Each biomarker in the panel is supported by experimental validation. Priority is given to molecules detectable in body fluids, such as blood or bile, which enhances feasibility and operational practicality in clinical workflows. Although these combinations have not yet been clinically validated as integrated panels, their underlying logic provides a conceptual foundation for future bioinformatics modeling and clinical testing.

Despite these advances, most multi-omics biomarkers in iCCA remain at the preclinical or mechanistic validation stage and have yet to enter routine clinical use. Several key challenges must be addressed to enable clinical translation. First, systematic validation across multi-center cohorts and ethnically diverse populations is required to establish stability and generalizability. Second, standardization of sample collection, processing, assay protocols, and data normalization is essential to ensure reproducibility and comparability across studies. Third, regulatory pathways—including biomarker qualification by the FDA or NMPA—should be considered early in development. Finally, integrating these biomarkers into clinical decision-making frameworks requires demonstrating prognostic or predictive value in well-characterized patient cohorts, along with alignment with feasible, standardized testing platforms. Addressing these challenges is crucial for advancing promising multi-omics biomarkers from research findings into actionable tools for precision oncology in iCCA [99].

Notably, recent research is shifting from static genomic and transcriptomic profiles toward identifying functional biomarkers that reflect dynamic molecular network activity. Functional biomarkers, such as phosphorylated FGFR (p-FGFR) and STAT3 (p-STAT3), provide a direct readout of signaling pathway activation and drug responsiveness [100]. Future multi-omics studies should expand into pathway activation, post-translational modifications, and ligand–receptor interactions to build an integrated, cross-layer functional biomarker system that is clinically operable.

At the same time, multi-omics–based molecular subtyping is emerging as a key strategy for precision stratification and therapy. Recent integrative analyses combining genomic, transcriptomic, proteomic, and phosphoproteomic data have identified three core molecular subtypes of iCCA—Proliferative, Metabolic, and Immune—each with distinct molecular signatures and therapeutic response profiles [68]. This represents a successful model of systematic multi-omics integration, revealing iCCA’s intrinsic heterogeneity while providing a practical framework for individualized treatment design.

In summary, future iCCA research should prioritize the integration of high-resolution multi-omics data, development of dynamic liquid biopsy-based biomarker detection platforms, and identification of novel therapeutic targets. Progressing these efforts through interdisciplinary collaboration and large-scale clinical validation will be critical to accelerate the translation of biomarker discoveries into clinical practice, enabling more precise and effective personalized treatment strategies for patients with iCCA.

## 6. Highlights

Biomarkers play a crucial role in the early diagnosis, prognostic assessment, and treatment decisions of intrahepatic cholangiocarcinoma

Advances in multi-omics technologies have significantly facilitated the identification of novel biomarkers

The clinical application of biomarkers is driving the development of precision medicine

## Figures and Tables

**Table 2:** Prognosis-related Transcriptomic Biomarkers in iCCA.

Prognostic Assessment	Gene Name	Category	Source	Mechanism	Ref.
High expression is correlated with unfavorable prognosis	MAL2	mRNA	tissue	Reprograms lipid metabolism via EGFR/SREBP-1 signaling in iCCA	[35]
	MFAP5	mRNA	serum, tissue	Activates the Notch1 signaling pathway to promote iCCA aggressiveness	[36]
	SPRY4-IT1	lncRNA	tissue	Upregulates in CCA and associates with advanced tumor stage and poor survival	[37]
	FAM66C	lncRNA	tissue	Drives tumorigenesis and glycolysis via the miR-23b-3p/KCND2 axis; enhances proliferation and metastasis	[37]
	MNX1-AS1	lncRNA	tissue	Promotes iCCA progression via the MNX1-AS1/c-Myc/MAZ/MNX1/Ajuba/Hippo signaling cascade	[38]
	ZEB1-AS1	lncRNA	tissue	Promotes cancer progression via the miR-200a/ZEB1 signaling pathway	[38]
	CASC15	lncRNA	tissue	Correlates with larger tumor size, advanced TNM stage, and poor prognosis	[39]
	circZNF215 (hsa_circ_0096334)	circRNA	tissue	Promotes tumor growth and metastasis by inactivating the PTEN/AKT pathway in iCCA	[40]
	circSLCO1B3 (hsa_circ_0025580)	circRNA	tissue	Promotes iCCA proliferation and metastasis via the miR-502-5p/HOXC8/SMAD3 axis and inhibits PD-L1 degradation via SPOP	[41]
	circPCNXL2 (has_circ_0016956)	circRNA	tissue	Promotes tumor growth and metastasis by interacting with STRAP to activate ERK signaling in iCCA	[42]
	circLTBP2 (has_circ_0032603)	circRNA	serum, tissue	Promotes pro-metastatic targets by competitively regulating miR-338-3p	[43]
	circHMGCS1-016 (hsa_circ_0008621)	circRNA	tissue	Induces immune evasion by sponging miR-1236-3p and upregulating CD73 and GAL-8	[44]
	circACTN4 (Hsa_circ_0050898)	circRNA	tissue	Promotes iCCA progression by recruiting YBX1 to activate FZD7 transcription	[45]
	circCCAC1 (hsa_circ_0043469)	circRNA	bile, tissue	Promotes iCCA by sequestering EZH2 in the cytoplasm, elevating SH3 domain-containing homolog	[46]
	miR-3191-5p	miRNA	tissue	Suppresses tumor growth via p53 activation and associates with better survival	[47]
Low expression is correlated with unfavorable prognosis	circUGP2 (hsa_circ_0001020)	circRNA	tissue	Suppresses iCCA progression via p53 signaling by targeting UHRF1 and regulating ADGRB1 transcription	[47]
	miR-338-3p	miRNA	tissue	Suppresses iCCA progression by targeting oncogenic transcripts and correlates with improved OS and RFS	[48]

**Table 3:** Proposed Multi-Omics Biomarker Panels for iCCA Diagnostic.

Panel	Biomarkers	Sample Source	Omics Source	Applicable Clinical Scenario	Rationale
**Blood-based screening panel**	Glutamine + MFAP5 + CA19-9 + CEA	Blood (serum/plasma)	Metabolomics + Transcriptomics + Proteomics	Early-stage screening, liquid biopsy	Easily accessible, biologically complementary, suitable for high-throughput diagnostic platforms
**Bile-based confirmation panel**	circCCAC1 + CDO1 methylation + SEPT9 methylation	Bile	Transcriptomics + Epigenetics	Confirmatory diagnosis in high-risk individuals	High specificity, applicable for PSC patients or inconclusive imaging cases via endoscopic sampling

**Table 4:** Recommended Combinations of Therapeutic Biomarkers and Clinical Application Potential.

Panel	Biomarkers	Omics Source	Mechanism Summary	Proposed Therapeutic Strategy	Applicable Clinical Scenario
**Metabolic-Mutation Panel**	IDH1/2 mutation + R-2HG accumulation + ALDH overexpression	Genomics + Metabolomics + Proteomics	Alters metabolic reprogramming, promotes malignant phenotype	mIDH1 inhibitors (e.g., Ivosidenib)+ ALDH inhibitors (e.g., NCT-501)	IDH-mutant or metabolically dysregulated iCCA subtypes
**Immune-Resistance Panel**	KRAS mutation + Treg infiltration + PD-1 overexpression	Genomics + Transcriptomics	Induces immunosuppressive TME, mediates poor immunotherapy response	KRAS inhibitors (e.g., sotorasib) combined with PD-1 checkpoint blockade	KRAS-mutant iCCA with immune-cold phenotype and resistance to PD-1 therapy
**Drug Resistance Targets**	MAL2, circLTBP2, circHMGCS1-016	Transcriptomics	Modulate sensitivity to cisplatin, gemcitabine, and PD-1 blockade	Combination strategies tailored by functional validation	iCCA patients with high risk of primary or acquired resistance

**Table 5:** Recommended Combinations of Prognostic Biomarkers.

Subtype	Omics Characteristics	Prognostic Biomarker	Prognostic Trend
**S1 Inflammatory subtype**	**Proteomics**: Upregulation of inflammatory proteins such as MPO, CD14, and C5AR1	SLC16A3	Worst
	**Genomics**: KRAS mutations		
	**Immunomics**: Infiltration of immunosuppressive cells; high expression of IDO and PD-L1		
**S2 Mesenchymal subtype**	**Proteomics**: High expression of CAF/ECM-related proteins such as POSTN and FAP	POSTN	Intermediate
	**Immunomics**: Enhanced angiogenic signaling; elevated expression of immune checkpoints such as CD276		
**S3 Metabolic subtype**	**Proteomics**: Upregulation of metabolic enzymes such as FASN, IDH1, and MAPK-related proteins	ALDOB	Favorable
	**Genomics**: TP53 mutations enriched		
	**Metabolomics**: Active carbon metabolism		
**S4 Differentiated subtype**	**Proteomics**: High expression of biliary differentiation markers such as EPCAM and KRT18	HKDC1	Best
	**Genomics**: Mutations in FGFR2, IDH1/2, and BAP1		
	**Immunomics**: Enriched CD8^+^ naive T cells		

## Data Availability

No new data were created or analyzed in this study. Data sharing is not applicable to this article.

## Abbreviations

Abbr	Full Name
CA19-9	carbohydrate antigen 19-9
CEA	carcinoembryonic antigen
CNV	copy number variations
dCCA	distal cholangiocarcinoma
ECM	extracellular matrix
FDA	the U.S. Food and Drug Administration
HCC	hepatocellular carcinoma
iCCA	intrahepatic cholangiocarcinoma
LC-MS/MS	liquid chromatography–tandem mass spectrometry
NIH	National Institutes of Health
pCCA	perihilar cholangiocarcinoma
scRNA-seq	single-cell RNA sequencing
SNP	single nucleotide polymorphisms
ST	spatial transcriptomics
WGBS	whole-genome bisulfite sequencing
